# Molecular Targets and Related Biologic Activities of Fucoidan: A Review

**DOI:** 10.3390/md18080376

**Published:** 2020-07-22

**Authors:** Zhen Lin, Xiaohui Tan, Yu Zhang, Fangping Li, Ping Luo, Huazhong Liu

**Affiliations:** Faculty of Chemistry & Environment Sciences, Guangdong Ocean University, Zhanjiang 524088, China; linz199771@163.com (Z.L.); tanxiaohuiii@163.com (X.T.); joanne96zy@163.com (Y.Z.); 15709482571@163.com (F.L.); luopingna@163.com (P.L.)

**Keywords:** fucoidan, molecular target, biologic activity

## Abstract

Fucoidan—a marine natural active polysaccharide derived from brown algae with a variety of medicinal activities and low toxicity—has been used as clinical drug for renal diseases for nearly 20 years. The pharmacological mechanism of fucoidan has been well-investigated, based on target molecules and downstream signaling pathways. This review summarizes some important molecular targets of fucoidan and its related biologic activities, including scavenger receptor (SR), Toll-like receptors (TLRs), C-type lectin (CLEC) and some newly found target molecules, which may be beneficial for further understanding the pharmacological mechanism of fucoidan and discovering its new functions, as well as developing related clinical or adjuvant drugs and functional preparations.

## 1. Introduction

Natural polysaccharides are sorts of important biologic macromolecules that possess a variety of biologic activities, including antibacterial, antioxidant, anti-inflammatory and antitumor, etc. In recent years, growing evidence supports potential of natural polysaccharides for the treatment and prevention of disease, due to their high-activity, low-toxicity and fewer side effects [1]. As a type of hydrophilic biologic macromolecules, polysaccharides cannot pass through cytomembranes freely, but instead bind to pattern-recognition receptors (PRRs), a group of membrane receptors including scavenger receptors (SRs), Toll-like receptors (TLRs), complement receptor 3 (CR3, aMb2-integrin, CD11b/CD18), C-type lectin receptors (CLRs), mannose receptor (MR) or other target molecules to trigger intracellular signaling cascades, mediating cellular physiological mechanisms, such as activating immune response [2,3].

**Figure 1:**
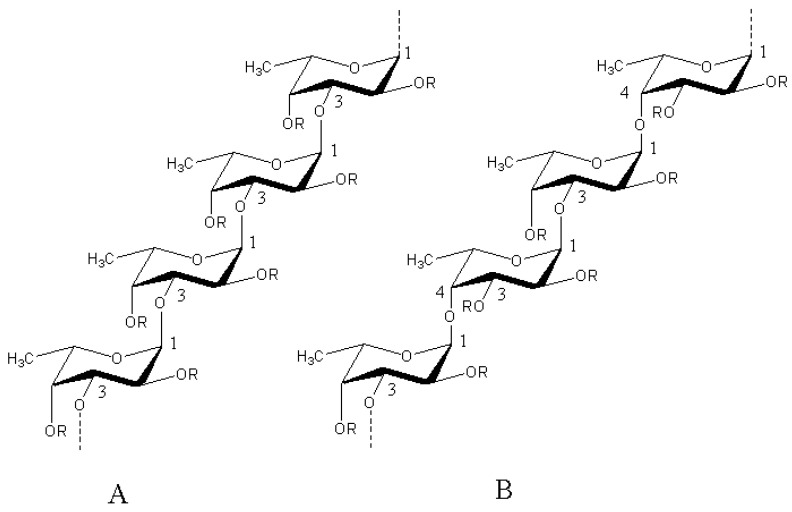
Two main types skeletal structure of fucoidan. Structure of type (**A**) fucoidan are constructed of only repeating (1→3)-linked α-l-fucopyranose residues whereas type (**B**) consist of alternating (1→3)- and (1→4)-linked α-l-fucopyranose residues. R represents the places of potential attachment of carbohydrate (α-l-fucopyranose, α-d-glucuronic acid) and noncarbohydrate (sulfate and acetyl groups) substituents [8].

Fucoidan is a kind of marine-sulfated polysaccharide derived from brown algae such as *Laminaria japonica*, *Sargassum cinereum*, *Fucus vesiculosus* [4,5,6]. Most differently originated fucoidans consist of sulfated L-fucose accounting for about 34–44%, small proportions of galactose, mannose, xylose and uronic acids, as well as acetyl groups and proteins [7]. The skeletal structure that composes fucoidan can be divided into two main types, (1→3)-linked α-l-fucopyranose residues and alternative combination of (1→3)- and (1→4)-linked α-l-fucopyranose residues (Figure 1) [8]. Some reviews have summarized the medicinal activity of fucoidan, but the pharmacological mechanism of fucoidan has not been described from the perspective of receptors or affinity molecules. In order to further explore the biologic activity of fucoidan and apply it widely in disease therapeutic strategies, it is inevitable to discuss which molecules upon which it targets its binding. Therefore, this review summarized some important achievements concerning molecular targets of fucoidan.

## 2. Molecular Targets of Fucoidan

### 2.1. Class A Scavenger Receptor

Goldstein and coworkers first, reported that fucoidan could bind to a low-density lipoprotein (LDL) recognition site that turned out to be class A scavenger receptor (SR-A) on macrophages [9]. SRs are a group of transmembrane proteins with homotrimeric structure, which preferentially express on macrophages and function in regulating lipid metabolism, atherosclerosis formation and many metabolic processes [10]. SRs recognize and internalize a variety of macromolecules and polyanionic compounds, such as LDL, lipopolysaccharide (LPS) and lipoteichoic acid (LTA).

SR-A, known as cluster of differentiation 204 (CD204), is a subclass of SRs and lacks typical signaling sequence in its cytoplasmic N-terminus. The broad range of polyanionic ligands can be recognized by SR-A and then triggers signaling cascades in macrophages, involved in macrophagic activation and inflammatory processes [11]. Due to the negative charge of fucoidan, it has been used as a common ligand to investigate SR-A involved biologic function and the molecular mechanisms in macrophages.

#### 2.1.1. Fucoidan/SR-A Involved Biological Functions and Related Molecular Mechanisms

SR-A is a widely distributed membrane receptor that has been found on many types of cells, including macrophages, renal tubular epithelial cells and mesenchymal cells, dendritic cells, endometrial cells, vascular smooth muscle cells and endothelial cells, etc., [10,12,13,14] which suggests that fucoidan possesses extensive biologic functions in diverse organs/tissues via binding to SR-A (Table 1). Noteworthily, most of the published researches focused on macrophages.

##### Cooperation between SR-A and Other Receptors

Previous work reported that fucoidan promoted expression of inducible nitric oxide synthase, urokinase-type plasminogen, interleukin 1, tumor necrosis factor α and interleukin 10 through SR-A in macrophages [15,16,17]. The mechanisms were closely related with some important protein kinases and mitogen-like signals, including p38 mitogen-activated protein kinase (MAPK), nuclear factor-kB (NF-κB) dependent pathways, protein kinase C (PKC), etc. [15,16,17].

**Figure 2:**
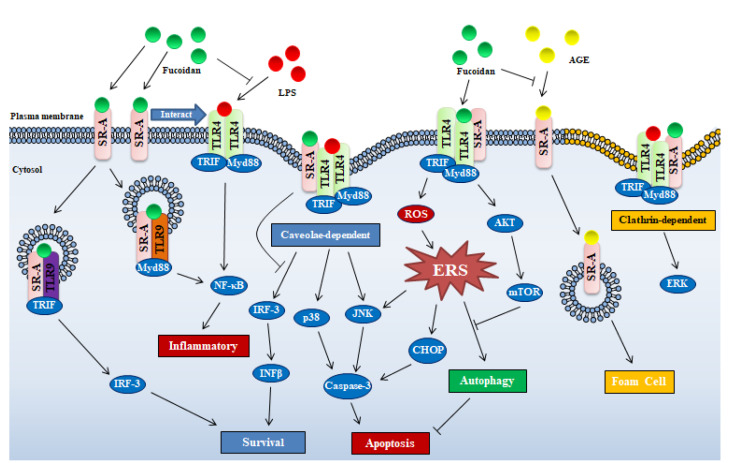
Schematic illustrating mechanisms of fucoidan-mediated intracellular signaling through SR-A and TLR-4. Abbreviations in figure: PERK—protein kinase R-like ER kinase; IFN-β—interferon-β; CHOP—C/EBP-homologous protein; mTOR—mammalian target of rapamycin; IRF-3—interferon regulatory factor 3; ROS—reactive oxygen species; AKT—protein kinase B.

Although SR-A has been demonstrated to trigger intracellular signaling cascades, it is not known how fucoidan/SR-A activates the signaling pathways, since the receptor has no typical signaling sequence in its cytoplasmic N-terminus. Many investigators speculatively attributed SR-A triggered intracellular signaling events to cooperation with other membrane receptors such as TLRs [10]. Seimon et al. discovered that fucoidan/SR-A triggered JNK-dependent apoptosis in endoplasmic reticulum of stressed peritoneal macrophages by cooperating with TLR4 to alter TLR4-signaling from pro-survival to pro-apoptotic [18]. Yu et al. revealed that SR-A was required for LPS-induced TLR4-mediated NF-κB activation in macrophages exposed to fucoidan [14], but SR-A was not involved in NF-κB gene expression [18]. These data suggest that although SR-A is a high-affinity receptor of fucoidan, fucoidan/SR-A-mediated intracellular signaling-cascades should be cooperative with TLR4 (Figure 2).

##### SR-A Mediated Internalization of Ligands

It is also known that SR-A is a primary endocytic receptor shown to internalize fucoidan [14]. TLR4 is a receptor that can be activated by fucoidan to trigger NF-κB-signaling pathway and consequent proinflammation [19]. However, anti-inflammation as a conclusive activity of fucoidan has been well-recognized. Hence, SR-A internalizing, deleting TLR4 ligand and consequently ameliorating activation of TLR4 should be an important reason for anti-inflammation, especially coexistence of inflammatory substance LPS. Pei et al. showed that, in rat microglia HAPI cells, fucoidan significantly inhibited the internalization of LPS, but failed to inhibit LPS-induced superoxide production, which suggested that fucoidan blocked internalization of LPS through binding to SR-A [20]. Similarly, fucoidan repressed SR-A internalizing polyanionic polypeptides into J774 cells [21]. Advanced glycation end-products (AGE) that derived from prolonged exposure of proteins to sugars is another kinds ligand of the SR-A. On account of the affinity to SR-A, fucoidan may be potential candidates for inhibitors of toxic AGE uptake, so the toxicity of AGE endocytosis can be attenuated by fucoidan (Figure 2) [22].

Factually, SR-A-mediated internalization of ligands is not merely a “passive” event, but an effective way to achieve signal diversification and specificity [11]. SR-A/ligand complex can be internalized by macrophage through two endocytosis ways, clathrin- and caveolae-dependent pathways, the latter way was required by SR-A/fucoidan internalization to trigger apoptosis, which was linked to p38 kinase and c-Jun-N-terminal kinase (JNK) activation (Figure 2) [11]. Similarly, under the action of SR-A ligand, SR-A interacts with major vault protein to activate the JNK-signaling pathway through the caveolin-mediated endocytic pathway [23].

##### SR-A Involved Endoplasmic Reticulum Stress Pathway

The endoplasmic reticulum stress (ERS) induced autophagy in macrophages was inhibited by fucoidan/SR-A through activating the AKT/mTOR/p70S6K pathway, which should be a critical reason for apoptosis induction of macrophages (Figure 2) [24]. Seimon et al. deduced that fucoidan induced apoptosis in macrophages with ERS via SR-A receptor required cooperation of TLR4 receptor [18]. The findings reveal the direct relationship between SR-A and ERS, however it is still unknown what mechanisms are used by fucoidan activated SR-A to regulate ERS, further investigation should be explored in the future.

Based on literature, effect of fucoidan on cellular function through SR-A can be divided into two topics, one is that fucoidan/SR-A mediates intracellular signaling events by interacting with other receptors or pathways (ERS and endocytosis also be involved), the other is that fucoidan competitively binds to SR-A to exclude effects of other ligands on cellular function (Figure 2). Despite the existence of many mechanisms, it is not hard to see that SR-A plays an important role in the regulation of immune function by fucoidan. Moreover, most studies on SR-A and fucoidan have focused on macrophages in vitro, however, SR-A has been described in vascular smooth muscle cells, endothelial cells, human lung epithelial cells, microglia, astrocytes and murine embryonic fibroblasts [10]. In other words, considering the distribution of SR-A alone, we can recognize that fucoidan has many functional sites in the body instead of being limited to immune cells. Equally important, as also described in the literature, it can be seen that fucoidan has complex regulation of intracellular signals through SR-A, so it is difficult to determine the role of fucoidan in disease by single in vitro experiments on certain cells.

### 2.2. Toll-Like Receptors

TLRs is a group of evolutionary conserved transmembrane proteins that recognize endogenous and exogenous ligands. Different from SR-A, TLRs contain N-terminal leucine-rich repeats and a C-terminal Toll/IL-1R homology domain, and can transmit signals downstream through the adapter proteins MyD88 (myeloid differentiation factor), MyD88 adapter-like, TIR-domain-containing adapter-inducing IFN-β (TRIF) and TRIF-related adaptor molecule [25]. To date, a total of 13 TLR species have been identified, including TLR1~TLR11 expressed in humans, which can be divided into two categories according to the different expression location. The first category distributes on the cell surface, such as TLR1, TLR2, TLR4, TLR5, TLR6 and TLR11, and the second category is located in cell, including TLR3, TLR7, TLR8 and TLR9 [26]. Different types of TLRs recognize different types of ligands, among which the ligands of TLR2 and TLR4 located on the surface of the cell membrane are lipids and lipid peptides, while the ligands of TLRs located in cell, such as TLR3, recognize nucleic acids [26].

Polysaccharides from natural sources can be recognized by TLR2 and TLR4, and the signal through NF-κB rapidly translocates from the cytoplasm to the nucleus and then regulates the expression of correspondent target genes, promoting release of proinflammatory cytokines, and thus regulating immune function [25,26]. Teruya et al. demonstrated that fucoidan can be recognized by TLR4 combined with SR-A and CD-14 that triggers MAPK-signaling pathways, resulting in NO production in macrophage [27]. Members of the MAPK family, p38 MAPK, stress-activated protein kinase/c-Jun-N-terminal kinase (SAPK/JNK) and extracellular signal-regulated kinase 1/2(ERK1/2), are downstream signaling pathways of TLR4 that can be activated by fucoidan, but ERK1/2 do not involve in induction of NO in macrophages (Table 1) [27].

Fucoidan from brown algae activates NF-κB through TLR-2 and TLR-4 on HEK293 cells, the TLRs had different affinity to differently originated fucoidan [19]. TLR4 activated by fucoidan induces ROS-associated ERS by activating the PERK–ATF4–CHOP pathway [28] and promotes caspase-3 activation through the TLR4–ERS–CHOP pathway, resulting in apoptosis in lung cancer cells (Figure 2; Table 1) [29].

It is reasonable that TLR activation mediates the synthesis and secretion of pro-inflammatory cytokines, but it is well-known that fucoidan possesses anti-inflammatory activity. For example, Park et al. showed that fucoidan exhibited anti-inflammatory properties by repressing LPS/TLR4 induced activation of NF-κB, ERK, JNK, p38 MAPK and AKT-signaling pathways [30]. The apparently contradictory results may be well-explained by fucoidan activated SR-A. Studies have shown that SR-A has a negative regulatory effect on TLR4-mediated immune cell activation [31]. Hence, cooperation between SR-A and TLR4 should be crucial for fucoidan associated bio-function (Figure 1; Table 1).

Additionally, TLR3 and TLR9 participate in fucoidan associated activity. Internalized SR-A interacts with TLR3 to promote cell survival via TLR3/IRF-3 pathway or with TLR9 to mediate cell to undergo inflammation or death via TLR9/TRIF/NF-κB/TNF-α (tumor necrosis factor-α) pathway [10], which suggests that fucoidan plays pro-survival or pro-death role via interaction between SR-A and TLR3/TLR9 (Figure 2).

### 2.3. C-Type Lectins (CLEC)

#### 2.3.1. C-Type Lectin Receptors (CLRs)

As a group of members of membrane-bound C-type lectins (CLEC), C-type lectin receptors (CLRs) are another group of members of PRRs family. CLRs not only recognize various antigens, but also transmit intercellular signals, assisting macrophages and dendritic cells to induce innate immunity [26,59]. CLRs contain dectin-1, dectin-2 cluster, mannose receptor and DC-specific ICAM-3 grabbing non-integrin [25]. These receptors can recognize components of fungal cell walls, such as β-glucans and mannose and fucoidan [32]. Fucoidan blocks cell adhesion via binding to Ly 49 family, member of CLRs [32,33]. Additionally, fucoidan also recognizes the osteoclast inhibitory lectin (OCIL) family that belongs to CLRs of NK cell, but the binding does not affect OCIL inhibition of osteoclast formation [34].

Recognition of the above-mentioned receptors on fucoidan is closely related to their carbohydrate recognition domain. However, in addition to the recognition of polysaccharides by ligand-binding domain, intracellular domain of CLR receptor also attributes to the species-specific ligand profile. The high-valency-glucan curdlan activates both human and mouse dectin-1, but the low-valency-glucan only activates cells expressing human dectin-1 and not mouse dectin-1 [60]. The reason can be attributed to the difference in amino acid composition of the human and mouse dectin-1 intracellular domain amino, leading to different sensitivity of the receptor [60].

#### 2.3.2. CLEC-2

**Figure 3:**
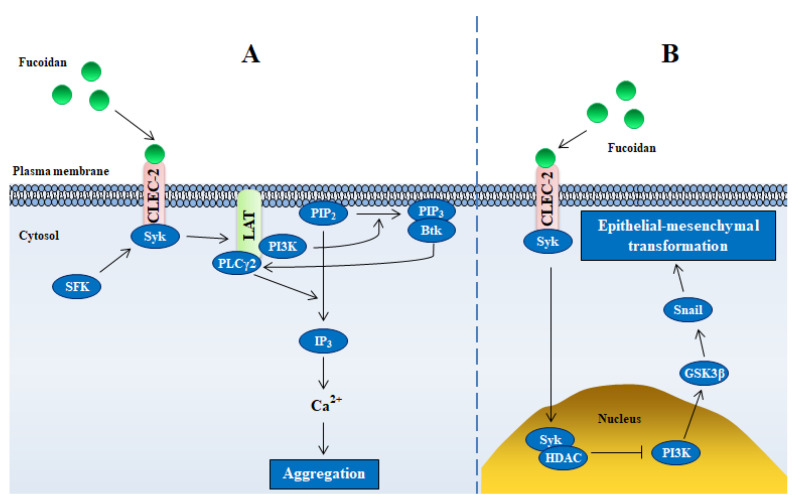
Schematic illustrating mechanisms of fucoidan-mediated intracellular signaling through CLEC-2. (**A**,**B**) represent platelet cells and gastric cancer cells, respectively. Abbreviations in figure: SFK—Src family kinases; Syk—spleen tyrosine kinase; PI3K—phosphoinositol 3 kinase; PIP_2(3)_—phosphoinositol 4,5(3)-bisphosphate (trisphosphate); PLCγ2—phospholipase Cγ2; Btk—Bruton’s tyrosine kinase; GSK3β—glycogen synthase kinase-3 beta; HDAC—histone deacetylase.

As transmembrane receptors mainly expressed in platelets, the superfamily of C-type lectin 2 (CLEC-2) can be activated to stimulate platelet aggregation by binding its phosphorylated atypical immunoreceptor tyrosine-based activation motif with tandem Src homology 2 domains of spleen tyrosine kinase [35]. The receptor was reported to be activated by fucoidan to mediate platelet aggregation through a tyrosine kinase-dependent signaling pathway, resulting in decrease of bleeding time in hemophilia animal models [36,37]. Activation of CLEC-2 by fucoidan phosphorylate PLCγ2 in the signalosome, leading to IP_3_ production sufficient for evoking oscillations of the cytosolic Ca^2+^ concentration and consequent platelet aggregation (Figure 3A) [38]. Another surface receptor of platelet, glycoprotein VI (GPVI) was also activated by fucoidan, which also contributed to stimulate platelet aggregation [36]. These reasons define the procoagulant property of fucoidan, but some studies reported anticoagulant activity of fucoidan. Martyanov et al. deemed that IP_3_ associated coagulant property is enough to overwhelm fucoidan’s anticoagulant activity [38]. Therefore, fucoidan should not be used as an alternative to low-molecular weight heparin [38].

In addition to CLEC-2-mediated platelet aggregation, fucoidan also targets CLEC-2 in tumor cells. In normal gastric mucosa, CLEC-2 is highly expressed, loss of CLEC-2 contributes to epithelial mesenchymal transformation and metastasis of gastric cancer (GC) (Figure 3B) [39]. Fucoidan binding to CLEC-2 increases the expression of CLEC-2 in GC cells by regulating CDX2 (caudal type homeobox transcription factor 2), an important regulator of gut homeostasis [35]. Moreover, fucoidan represses transforming growth factor-β1 secretion in different GC cells, leading to inhibition of cell growth, migration and invasion, which could be restored by knocking down CLEC-2 [35]. These results suggest that CLEC-2 is a potential target of fucoidan for the treatment of gastric cancer.

#### 2.3.3. Selectins

Fucoidan is also recognized by another type of proteins in the CLEC family, selectins, vascular cell adhesion molecules. Selectins are identified as L-selectin expressed on leukocytes, E-selectin expressed on endothelial cells and *P*-selectin expressed on platelet and endothelial cells (Table 1). Selectins mainly function in mediating leukocyte recruitment to sites of inflammation or to lymphoid tissues [40]. Fucoidan can block L- and *P*-selectins to delete endothelial-leukocyte interactions, resulting in better recovery of left ventricular function, coronary blood flow and myocardial oxygen consumption after cold ischemia [41]. Fucoidan improved coronary flow reserve and attenuated microvascular platelet deposition and platelet-mediated myocardial injury after transient ischemia through targeting *P*-selectins in a swine model of transient, thrombotic coronary occlusion [43]. Fucoidan substantially inhibited leukocyte urokinase plasminogen activator receptors-mediated Ca^2+^ mobilization through binding to the carbohydrate binding domain of L-selectin, which indicates interactions between selectins and other membrane receptors [42].

With more understanding of the combination of fucoidan and *P*-selectin, it is uncovered that the intensity of fucoidan binding to platelets is dependent on the level of platelet activation and that low molecular weight of fucoidan had an affinity for *P*-selectin at least two orders of magnitude higher than heparin and dextran sulfate [44]. Moreover, according to the binding characteristics between fucoidan and *P*-selectin, Rouzet et al. revealed that ^99m^Tc-fucoidan was a relevant imaging agent for in vivo detection of biologic activities associated with *P*-selectin overexpression, such as arterial thrombus and ischemic memory [45].

### 2.4. Other Affinity Molecules of Fucoidan

#### 2.4.1. Integrins: CR3 and αVβ3

The β_2_ integrin, CR3 (CD11b/CD18), is unique among integrins containing a carbohydrate binding lectin-like domain [46]. CR3 also contains another distinct ligand-binding site, I-domain which binds canonical ligands including extracellular matrix proteins, the complement component iC3b and intercellular adhesion molecules such as intercellular adhesion molecule 1 (ICAM-1) [46]. Studies showed that some natural plant polysaccharides activated immune cells through CR3 [47,48], such as fucoidan. Zen et al. reported that fucoidan inhibited adhesion of T84 cells to CD11b/CD18, the inhibitory activity of fucoidan was superior to heparin/heparin sulfate, *N*-acetyl-d-glucosamine, mannose-6-phosphate and laminarin [49]. Fucoidan directly bound to CD11b/CD18 in a divalent cation- and sulfation-dependent fashion could be blocked by anti-CD11b monoclonal antibodies (Table 1)[49]. CR3 is a critical receptor involved in NK cell activation by another sulphated polysaccharide that sulfated fucan (SF) extracted from *Stichopus japonicas*, moreover, protein and sulfate of SF are pivotal for the interaction between the SF and NK cells [61].

Apart from CR3, αVβ3 has been demonstrated to be another fucoidan receptor, an integrin that can be activated by fucoidan to mediate the Src/cortactin/E2F1-signaling pathway, functioning in anti-metastasis of SMMC-7721, Huh7 and HCCLM3 liver cancer cells (Table 1) [50].

#### 2.4.2. VEGF

Vascular endothelial growth factors (VEGF) have been considered as the targets to inhibit deregulated blood vessel formation, which influences endothelial cell proliferation, migration, invasion and vascularization [51,52]. Interaction between VEGF receptor 2 (VEGFR2) and VEGF can be disrupted by fucoidan through binding to both VEGF and VEGFR2, inactivating VEGFR2/Erk/VEGF-signaling pathway in HMEC-1 cells [51]. VEGF recognizes sulfated groups of fucoidan to impede VEGF/VEGFR2 interaction, thus affecting the downstream signaling molecules including Src family kinase, focal adhesion kinase and AKT kinase (Table 1) [52].

#### 2.4.3. CXCL12/CXCR4

C–X–C motif chemokine 12 (CXCL12) and C–X–C motif chemokine receptor type 4 (CXCR4) play a pivotal role in tumor growth, metastasis, cancer cell–microenvironment interactions as well as therapeutic resistance [53]. Tino et al. presented that fucoidan bound to CXCL12 and interfered the CXCL12/CXCR4 axis in human Burkitt’s lymphoma cells, thereby blocking both CXCL12-induced CXCR4 receptor activation and downstream effects, such as migration and matrix metalloproteinase-9 secretion (Table 1) [53].

#### 2.4.4. Elastin Peptide Receptor

The potential target of fucoidan for regulating immune function is not limited to the PRRs mentioned in the above sections. Fucoidan binds to elastin peptide receptor of monocytes, recruiting monocytes in vitro and in vivo (Table 1) [54]. Interestingly, a polysaccharide from pathogenic fungus, *Candida albicans*, has similar chemoattracting properties to fucoidan, even though the concentration is lower. Thereby Li et al. speculated that the chemotactic response of monocytes to the sulfated fucogalactan is part of the innate immune system to fungal infection [54].

#### 2.4.5. TGF-β1

Transforming growth factor-β (TGF-β) is a family of proteins that exert diverse and potent effects on proliferation, differentiation and extracellular matrix synthesis [56]. TGF-β1 is the representative of fibrogenic cytokines, which is secreted from cells as a small latent TGF-β1 (LTGF-β1) complex or more commonly as a large LTGF-β1 complex, TGF-β1 has to be cleaved from latency associated peptide of the latent complex to be active [57]. Potential anti-fibrotic mechanism of fucoidan is that TGF-β1 bound to fucoidan is unable to interact with its receptor, thereby attenuating phosphorylation of Smad2 which forms heteromeric complexes with Smad3 and Smad4, resulting in transcription inhibition of the target genes, including fibronectin and collagen (Table 1) [57]. Moreover, fucoidan also binds to LTGF-β1 and inhibits LTGF-β1 activation, the inhibitory effects on TGF-β1 and LTGF-β1 are positively related to molecular weight of fucoidan [57].

#### 2.4.6. ECM Proteins

In terms of anti-cell adhesion, fucoidan not only targets cell surface receptors, also binds to several extracellular matrix (ECM) proteins, among which the binding of cancer cells to ECM proteins is considered to be a fundamental step in the progression of metastasis. Rocha et al. showed that fucoidan inhibited both wild-type and mutant CHO cell adhesion to fibronectin (FN), an EMC protein, through binding directly to FN, thus blocking FN sites that can be recognized by cell surface ligands, possibly the integrin family (Table 1) [58]. Fucoidan exhibited the highest inhibitory effect in comparison to other sulfated polysaccharides including heparin sulfate, heparin, dermatan sulfate, chondroitin 4-sulfate and chondroitin 6-sulfate, it is worth noting that this effect was abolished by desulfation of fucoidan [58].

## 3. Conclusions

Fucoidan is a type of sulfated and heterogeneous polysaccharide in the cell-wall matrix of various brown seaweed species. Fucoidan possesses antitumor, antivirus, antioxidation, antithrombotic, coagulant and anticoagulant, anti-inflammatory, immunomodulatory, as well as effects against various renal, hepatic and uropathic disorders. The medicinal preparation of fucoidan—Haikun Shenxi capsules—received a New Drug Certificate as a traditional Chinese medicine in 2003 from the Chinese Food and Drug Administration (CFDA). This medicine has been used as clinical drug of renal diseases for nearly 20 years in China [62]. This review focuses on explaining pharmacological mechanisms of fucoidan based on molecular targets. For instance, fucoidan targets SR-A to play immunomodulatory role via mediating internalization of ligands, cooperation with other receptors and ERS pathway. Fucoidan activates TLRs to perform antioxidation and immunoregulation, binds to CLEC to regulate immune function, coagulation, antitumor, etc. In addition, many important membrane-bound and non-membrane-bound proteins, such as integrins (CR3 and αVβ3), VEGF, CXCL12/CXCR4, elastin peptide receptor, TGF-β1 and some ECM proteins, are also regarded as target molecules by fucoidan to perform its various biologic activities.

At the end of the 19th century, Langley came up with the concept of receptor while describing the mechanism by which nicotine drives muscle contractions [63]. With the deepening of research, the receptor theory was gradually improved and become an important model for understanding the biologic activity of drugs. The exploration of active substances targets is not only of positive significance for understanding the nature of their therapeutic effect, but also important for drug modification and drug strategy. In recent years, part of the research focuses on the effects of fucoidan on organism growth, metabolism and related signaling pathways. However, the molecular targets interacted with fucoidan which is responsible for initiating a biologic response is often neglected. In the following studies, attention should be paid to the mechanism of fucoidan acting on that particular molecular target and the crosstalk of downstream signaling pathways.

## Figures and Tables

**Table 1:** Distribution of molecular targets and related biologic activity interacting with fucoidan.

Molecular Targets	Cell Types	Biologic Activity of Interaction with Fucoidan	Reference
Scavenger receptors			
SR-A	Macrophages, endothelial cells, lung epithelial cells, microglia, astrocytes, primary murine fibroblasts	Cooperative with Toll-like receptors 4 (TLR4), mediating intracellular signaling cascades Internalization of fucoidan Crosstalk with endoplasmic reticulum stress (ERS) pathway, promoting cancer cells apoptosis	[10,11,12,13,14,15,16,17,18,19,20,21,22,23,24]
Toll-like receptors			
TLR2	Macrophages, monocytes, dendritic cells, mast cells, neutrophils, natural killer (NK) cells fibroblasts, embryonic kidney cells	Activating the nuclear factor-κB (NF-κB)	[19,25,26]
TLR4	Macrophages, monocytes, dendritic cells mast cells, neutrophils, b lymphocytes, intestinal epithelium cells, cardiomyocytes, renal tubular epithelial and endothelial cells, podocytes, Kupffer cells, lung cancer cells	Activating NF-κB and mitogen-activated protein kinase (MAPK) signaling pathways Induces reactive oxygen species (ROS) associated ERS, resulting in cancer cells apoptosis	[18,19,25,26,27,28,29,30,31]
C-type lectins			
Ly-49	NK cells, subset of T lymphocytes	Blocking cell adhesion	[32,33]
OCIL	Macrophages, epithelial cells, mesenchymal cells, dendritic cells, lymphocytes	Cannot affect osteoclast inhibitory lectin (OCIL) inhibition of osteoclast formation	[34]
CLEC-2	Platelets, (low levels in immune cells), gastric epithelial cells	Activating tyrosine kinase-dependent signaling pathway, resulting in platelet aggregation Preventing expression of phosphoinositol 3 kinase (PI3K) subunits in a spleen tyrosine kinase (Syk)) dependent manner and suppresses metastasis of gastric cancer cells	[35,36,37,38,39]
L-selectin	Monocytes, dendritic cells, neutrophils, NK cells, B lymphocytes, T lymphocytes	Deleting endothelial-leukocyte interactions Inhibiting leukocyte urokinase plasminogen activator receptors-mediated Ca^2+^ mobilization	[40,41,42]
*P*-selectin	Platelets, endothelial cell, Kupffer cells	Attenuating microvascular platelet deposition and platelet-mediated myocardial injury	[40,41,43,44,45]
Other affinity molecules of fucoidan			
CR3	Macrophages, monocytes, neutrophils, NK cells	Inhibiting adhesion of T84 cells to complement receptor 3 (CR3)	[46,47,48,49]
αVβ3	Cancer cells, rapidly dividing endothelial cells	Suppressing metastasis of gastric cancer cells though Src/cortactin/ E2F transcription factor 1 (E2F1) signaling pathway	[50]
VEGF	N/A	Impeding vascular endothelial growth factors (VEGF)–VEGF receptor 2 (VEGFR2) interaction	[51,52]
CXCL12/CXCR4	N/A	Blocking both C–X–C motif chemokine 12 (CXCL12) induced C–X–C motif chemokine receptor type 4 (CXCR4) activation	[53]
Elastin peptide receptor	Mesenchymal cells, vascular smooth muscle cells, skin fibroblasts	Recruiting monocytes through binding to elastin peptide receptor of monocytes	[54,55]
TGF-β1	N/A	Exerting functions of anti-fibrosis through blocking transforming growth factor-β (TGF-β1) interacted with its receptor	[56,57]
ECM proteins	N/A	Suppressing metastasis of cancer cells though blocking extracellular matrix (ECM) proteins	[58]
